# Non-invasive assessment of intracranial compliance in idiopathic intracranial hypertension: an MRI-ICP study

**DOI:** 10.1038/s41433-024-03547-7

**Published:** 2025-01-24

**Authors:** Matilde Sassani, James L. Mitchell, Andreas Yiangou, Nigel Davies, Vijay Sawlani, Susan P. Mollan, Mark E. Wagshul, Alexandra J. Sinclair

**Affiliations:** 1 Department of Metabolism and Systems Science, College of Medicine and Health, University of Birmingham, Birmingham, B15 2TT UK; 2 Centre for Endocrinology, Diabetes and Metabolism, Birmingham Health Partners, Birmingham, B15 2TH UK; 3 Department of Neurology, University Hospitals Birmingham NHS Foundation Trust, Queen Elizabeth Hospital, Birmingham, B15 2WB UK; 4 RRPPS, Department of Medical Physics, University Hospitals Birmingham NHS Foundation Trust, Birmingham, B30 3HP UK; 5 Department of Radiology, University Hospitals Birmingham NHS Foundation Trust, Queen Elizabeth Hospital, Birmingham, B15 2WB UK; 6 Birmingham Neuro-Ophthalmology Unit, University Hospitals Birmingham NHS Foundation Trust, Birmingham, B15 2TH UK; 7 Gruss Magnetic Resonance Research Center, Albert Einstein College of Medicine, Bronx, NY USA

**Keywords:** Eye diseases, Biomarkers

## Abstract

**Background/objectives:**

Idiopathic intracranial hypertension (IIH) is a disease which threatens vision and causes disabling headaches, affecting women of childbearing age with obesity. It is characterised by raised intracranial pressure (ICP), measured invasively either with lumbar punctures or intracranially-inserted monitors. There is an unmet clinical need to develop non-invasive means to assess ICP. This study aims to utilise the MRI-ICP imaging technique to measure intracranial compliance index and assess its suitability as surrogate biomarker of ICP.

**Subjects/methods:**

Nine IIH patients and ten age, sex, and body mass index matched healthy controls were recruited. All participants underwent lumbar puncture, visual assessments, detailed headache phenotyping, and MRI-ICP scans to calculate intracranial compliance index at baseline. Following treatment, patients were invited to attend a one-year visit when all assessments were repeated.

**Results:**

There was significant (*p* = 0.017) reduction in intracranial compliance index in IIH (mean = 1006.0 cc/mmHg/cm, SD = ± 384.6 cc/mmHg/cm) compared to controls (mean = 1493.0 cc/mmHg/cm, SD = ± 411.8 cc/mmHg/cm), inversely correlating with lumbar puncture opening pressure (r = −0.502, *p* = 0.029). A significant inverse correlation between compliance index and headache disability was also found (r = −0.458, *p* = 0.049) and a trend for an association between lower compliance index and increased frequency of headaches (r = −0.430, *p* = 0.066). This latter became significant (*p* = 0.018) after accounting for use of analgesics. Following successful treatment, compliance index was increased in all patients at one year (mean of differences = 380.7 cc/mmHg/cm, *p* = 0.031).

**Conclusions:**

This is the first study to apply the MRI-ICP technique longitudinally in IIH. It illustrates reduced intracranial compliance index in IIH, correlating with opening pressure and headache disability and ameliorating with treatment.

## Introduction

Idiopathic intracranial hypertension (IIH or pseudotumor cerebri) is a disabling disease disproportionally affecting women of childbearing age living in socioeconomically deprived areas [1–3]. It is as a complex neuro-metabolic condition with weight being the main modifiable risk factor [4], hence, the incidence of IIH is increasing concurrently with the obesity epidemic [5] and weight loss is the mainstay of therapy [6, 7]. Clinical semiology comprises symptoms secondary to raised intracranial pressure (ICP): incapacitating headaches [8, 9], visual symptoms and papilloedema [6], and pulsatile tinnitus. In addition, there is emerging evidence for alterations of cognitive function [10], metabolic dysfunctions [11], increased risk of cardiovascular events [3], reduced fertility and pregnancy complications [12, 13]. Diagnosis of typical IIH requires papilloedema and raised ICP in the absence of secondary causes [14]. Intracranial pressure is assessed with lumbar puncture (LP) at diagnosis, whereas invasive intracranial ICP monitoring may be required in complex cases [15, 16].

There is an unmet clinical need to develop non-invasive techniques to measure ICP and advanced magnetic resonance imaging (MRI) could have potential. However, in clinical practice, imaging is solely employed to exclude pathologies causing increased ICP such as sinus venous thrombosis, hydrocephalus, space-occupying lesions or imaging findings suggestive of meningitis [6]. On anatomical MRI, there are recognised indicators of elevated ICP: changes in venous sinuses calibre, stenosis of transverse sinus, empty or partially empty sella turcica, and volumetric alterations of the optic nerve, including optic nerve tortuosity, optic nerve sheath distension and posterior optic globe-sclera flattening [17]. These findings, when present, increase the a priori probability of IIH, but are not diagnostic, nor are they used to monitor disease or for prognostication [17, 18].

**Fig. 1:**
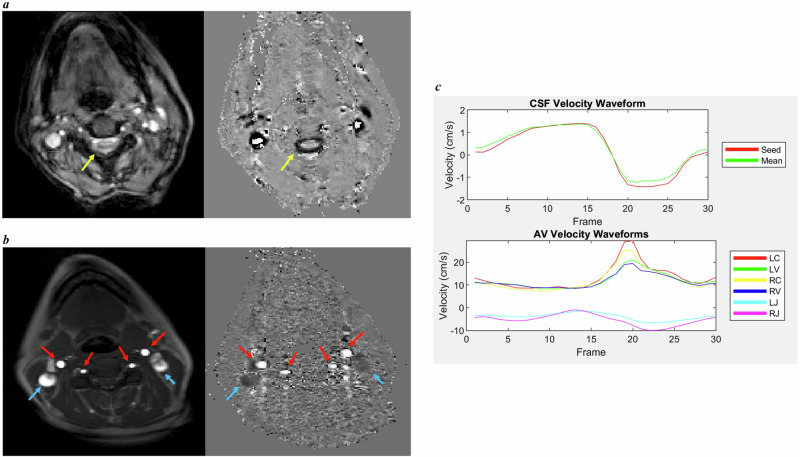
Images and waveforms used to calculate MRI-ICP parameters. **a** Magnitude (left) and phase (right) images acquired with low venc cine phase-contrast sequences utilised for calculations of cerebrospinal fluid (yellow arrows) flow parameters. **b** Magnitude (left) and phase (right) images acquired with high venc cine phase-contrast sequence utilised for calculation of arterial (red arrows) and venous (blue arrows) flow parameters. **c** Illustration of CSF (upper panel), arterial and venous (lower panel) waveforms; LC left carotid artery, RC right carotid artery, LV left vertebral artery, RV right vertebral artery, LJ left jugular vein, RJ right jugular vein.

MRI-ICP is an advanced quantitative MRI technique first described 20 years ago [19]. It utilises phase-contrast sequences to measure flow of internal carotid arteries, vertebral arteries, internal jugular veins, and cerebrospinal fluid (CSF). Arterial, venous and CSF waveforms are then calculated and used as direct measures of the change in intracranial volume over the cardiac cycle associated with these flows (Fig. 1). While changes in intracranial pressure cannot be measured directly, the time derivative of the CSF flow waveform at cranio-cervical junction can be used as an index of the change in the spatial pressure gradient over time (via the Navier-Stokes equation [20]). Taken together, these two measurements can be used to estimate intracranial compliance index (dV/dP), as well as stroke volumes and pressure changes associated with the cardiac-cycle [19]. Use of phase-contrast MRI to estimate ICP is still debated and being explored by the community. For instance, individual parameters, such as CSF flow velocity, have been shown to have a non-linear relationship with ICP [21]. A recent study utilised this non-linear association to estimate ICP in a large cohort of patients with communicating hydrocephalus, showing 86.1% accuracy [22]. Others have also assessed repeatability of phase-contrast MRI [23]. Craniospinal compliance, measured by MRI-ICP, has also been shown to have an inverse association with invasively measured ICP in patients with traumatic brain injury and elderly controls [24].

The aim of this study was to evaluate the MRI-ICP technique in women with active IIH at baseline and after having received treatment. Specifically, it was hypothesised that intracranial compliance index measured by MRI-ICP would be significantly different in patients with IIH compared to age and body mass index (BMI) matched women without IIH. In addition, it was hypothesised that there would be an inverse linear relationship between LP opening pressure and intracranial compliance index measured by MRI-ICP, that intracranial compliance index would correlate with clinical measures, and that it would improve with successful treatment.

## Methods

This case-control and prospective longitudinal radiological study was approved by the National Research Ethics Committee (West Midlands – The Black Country IIH:WT – 14/WM/0011 – ISRCTN trial registration: ISRCTN40152829) and conducted at the University of Birmingham and Queen Elizabeth Hospital, Birmingham, between August 2016 and September 2018. It was a sub-study of the randomised controlled and parallel-group IIH:WT trial (registered at https://clinicaltrials.gov/ct2/show/NCT02124486) which compared efficacy of surgical intervention versus community weight loss programme for treatment of IIH and the methodology has previously been published [25, 26].

Participants underwent clinical assessments and MRI at baseline. Patients were then randomised either to the bariatric surgery or a community weight loss programme arm according to published protocol [26]. Following the intervention, they were then invited to attend a follow-up research visit at one year.

### Research participants

Eligible patients who consented to this imaging research study were women between 18 and 55 year old, with a BMI ≥ 35 kg/m^2^ and a diagnosis of active IIH according to the revised diagnostic criteria (papilloedema with a Frisén grade ≥1 in at least one eye and opening pressure on lumbar puncture ≥25 cmCSF, equivalent to 18.4 mmHg) [14]. Inclusion criteria for the controls were: female sex, age 18–55 years, and BMI ≥ 35 kg/m^2^. Exclusion criteria for all participants were: pregnancy, inability to provide informed consent or contraindications to MRI. IIH patients were also excluded if they had had previous optic nerve sheath fenestration or previous bariatric surgery. Additional exclusion criteria for controls were: a diagnosis of IIH or LP opening pressure ≥25 cmCSF (i.e. ≥18.4 mmHg). Complete inclusion and exclusion criteria have been published [26]. Sample size was chosen to be analogous to other studies utilising phase contrast MRI in IIH [27, 28].

Patients were recruited from five National Health Care centres across the United Kingdom, whereas controls were recruited via advertisements on social media. Written informed consent was acquired from all participants according to the declaration of Helsinki.

### Visual and clinical parameters

At each visit, demographic data were acquired, and all participants underwent a lumbar puncture, visual testing, detailed headache phenotyping, and MRI.

Lumbar punctures were conducted in the lateral decubitus position with ultrasound marking guidance to measure opening and closing CSF pressures. Pressure was measured in cmCSF and then converted in mmHg using a 1.36 conversion factor to facilitate comparison with MRI results, also expressed in mmHg [29]. The LPs were undertaken after the visual and MRI measurements, to prevent the procedure from potentially affecting clinical and radiological results.

Papilloedema was graded by expert neuro-ophthalmologists using the Frisén scale [30] and quantified using the average peripapillary retinal nerve fibre layer (RNFL) with optical coherence tomography imaging (Spectralis^TM^, Heidelberg Engineering, Germany). Humphrey visual perimetry (Humphrey 24-2 Swedish Interactive Thresholding Algorithm central automated perimetry) was performed to calculate the perimetric mean deviation.

Headache burden was estimated through the 6-item headache impact test (HIT-6) questionnaire [31]. Participants also completed a daily headache diary to calculate number of monthly headaches (reported as headache days/month), average headache severity (scored on a scale from 0 to 10 with 10 as the most severe), average headache duration, and number of days of analgesic use per month. A physician with relevant specialist training in headache phenotyping characterised the participants’ headaches according to the 3^rd^ edition of the International Classification of Headache Disorders [32].

### Magnetic resonance imaging

All MRI scans took place using a 3T Siemens Verio (Siemens Healthineers, Erlangen, Germany) scanner using a 12-element Head Matrix with 4-element Neck Matrix attachment radiofrequency coil (Siemens Healthineers, Erlangen, Germany).

High-resolution 3D T_1_-weighted images were first acquired to aid accurate positioning of the phase-contrast flow sequences (sagittal orientation, echo time = 2.67 ms, repetition time = 1700 ms, inversion time = 900 ms, flip angle = 9°, field of view = 256 × 232 mm^2^, matrix = 256 × 232, voxel size = 1 × 1 × 1 mm^3^).

All velocity encoded cine phase-contrast sequences were acquired with retrospective cardiac gating utilising the following parameters: field of view = 200 × 200 mm^2^, matrix = 192 × 192 (not interpolated), slice thickness = 4 mm, bandwidth = 80 Hz/pixel, and 1 signal average. The CSF flow measurements were acquired axially at the level of C2-C3 intervertebral space, with echo time = 8.95 ms, repetition time = 40.15 ms, flip angle = 10°, and venc = 7 cm/s (increased to 15 cm/s in case of aliasing of the CSF area). The vascular sequences were acquired axially at the level of the cervical segment of the internal carotid artery with echo time = 5.05 ms, repetition time = 32.35 ms, flip angle = 30°, and venc = 70 cm/s (if aliasing of the vascular structures was present, the venc was incrementally increased by 20 cm/s until the artefact resolved).

Data analysis of the phase-contrast sequences was conducted according to previously published methodology [19, 33–35]. In brief, analysis was conducted by a researcher blinded to participants’ status with an in-house MATLAB code (version 9.13.0.2126072, R2022b, Natick, Massachusetts: The MathWorks Inc.) within a custom graphical user interface to visualise all images, calculate waveforms, and extract measurements. The technique measures net flow into and out of the cranium, hence it provides a measure of global compliance within the cranial cavity, and, as such, geometrical information intrinsic to the spinal canal or the cranium is not relevant. We used the pulsatility-based segmentation method [34] to limit calculations to regions of pulsatile flow: using the magnitude image of the phase-contrast sequences, the user defined seed points to attribute to CSF within the subarachnoid space, arterial cranial inflow (internal carotids and vertebral arteries) and venous outflow (internal jugular veins), Fig. 1. For each seed, the algorithm identifies the remainder portion of surrounding flow spaces by calculating the cross correlation between the seed and all other points within a pre-defined radius. These flow calculations were limited to eight-connected regions with a minimum cross correlation of 0.8. The final flow mask produced with this procedure was reviewed in all cases and edited if needed (e.g. if non-CSF/vessel regions were included or regions with flow were excluded). In addition, mean flow waveforms for arterial, venous and CSF compartment were reviewed in all cases to ensure biological plausibility: e.g. arterial and venous flow having opposite directions, and the trough of the venous waveform following the peak of the arterial waveform. Study parameters were then calculated from waveforms (i.e. intracranial compliance index, pressure gradient, and stroke volumes) as described [19]. Viscosity of CSF was not measured because the viscosity term of the Navier-Stokes equation is typically considered to be negligible [19] and CSF in IIH is, by definition, of normal composition [6]. Pressure gradients were calculated from pressure waves using four-point central difference formula to calculate the derivative at each time point.

Intracranial compliance index was the main parameter of interest, however, to allow comparison with literature, we also report the pressure gradient, as well as CSF, arterial, and venous stroke volumes.

### Statistical analysis

Between-group comparisons were tested either with unpaired two-tailed t-tests or Mann–Whitney tests depending on normality of distribution assessed using the D’Agostino and Pearson test [36]. Longitudinal data were compared with paired two-tailed t-tests. Simple linear regressions were used to test for correlations between compliance index and LP opening pressure, visual (RNFL) and headache measures (HIT-6, headache frequency, severity, and duration). In addition, to account for analgesics use as a potential confounder, multiple linear regressions were conducted entering in turn each headache parameter as the dependent variable and using compliance index and number of days of analgesic use per month as independent variables. We also used simple linear regression to test for associations between MRI-pressure gradient and LP opening pressure.

Depending on normality, mean and standard deviation (SD) or median and range are reported. For regressions, we report 95% confidence intervals, Pearson’s r, R^2^, variance inflation factors (VIF), and *p* values. Statistical significance was set at *p* < 0.05.

Statistical analysis was conducted using GraphPad Prism 9 v9.5.1 for Windows (GraphPad Software, San Diego, California USA, www.graphpad.com).

## Results

### Clinical characteristics at baseline

Nineteen women (ten controls and nine patients with active IIH) were included in this study. Patients and controls were well matched for age and BMI (Table 1), with a median interval between diagnosis and baseline research visit being 7.2 months. Table 1 reports clinical parameters.

**Table 1:** Clinical characteristics of study population at baseline and at one-year visit.

Cross-sectional: patients and controls						95% confidence interval	*p*
Age	Controls	Mean (±SD), *n*	40.1	(±10.8),	10	−15.36 to 4.05	0.236
	Patients	Mean (±SD), *n*	34.4	(±9.0),	9		
Body mass index (kg/m^2^)	Controls	Mean (±SD), *n*	45.5	(±5.3),	10	−9.23 to 7.47	0.826
	Patients	Mean (±SD), *n*	44.6	(±11.2),	9		
Opening pressure (mmHg)	Controls	Median (range), *n*	16.2	(7.7),	10	NA	**<0.0001**
	Patients	Median (range), *n*	22.1	(7.4),	9		
Closing pressure (mmHg)	Controls	Mean (±SD), *n*	11.3	(±1.7),	10	1.05 to 4.32	**0.003**
	Patients	Mean (±SD), *n*	14.0	(±1.7),	9		
Visual parameters							
Friesen grade	Patients	Mean (±SD), *n*	1.7	(±0.7),	10	NA	NA
Mean RNFL worst eye (µm)	Controls	Mean (±SD), *n*	99.2	(±7.8),	10	−8.56 to 43.05	0.177
	Patients	Mean (±SD), *n*	116.4	(±37.9),	9		
MD worst eye	Controls	Median (range), *n*	−2.3	(18.1),	10	NA	0.905
	Patients	Median (range), *n*	−2.4	(11.0),	9		
Headache parameters							
Headache disability (HIT-6)	Controls	Median (range), *n*	56.0	(30.0),	10	NA	0.055
	Patients	Median (range), *n*	65.0	(34.0),	9		
Monthly headache frequency	Controls	Median (range), *n*	12.0	(28.0),	10	NA	**0.044**
	Patients	Median (range), *n*	24.0	(28.0),	9		
Headache severity	Controls	Mean (±SD), *n*	3.4	(±2.7),	10	−1.65 to 2.98	0.552
	Patients	Mean (±SD), *n*	4.1	(±2.1),	9		
Headache duration (hours)	Controls	Median (range), *n*	2.4	(12.1),	10	NA	0.284
	Patients	Median (range), *n*	5.0	(24.0),	9		
Analgesic use per months (days)	Controls	Mean (±SD), *n*	7.1	(±7.4),	9	−6.01 to 13.12	0.442
	Patients	Mean (±SD), *n*	10.7	(±11.3),	9		
LONGITUDINAL: PATIENTS							
Body mass index (kg/m^2^)	Baseline	Mean (±SD), *n*	45.4	(±12.5),	6	−13.37 to 2.04	0.117
	One year	Mean (±SD), *n*	39.7	(±18.2),	6		
Opening pressure (mmHg)	Baseline	Mean (±SD), *n*	23.1	(±2.1),	6	−11.51 to -0.25	**0.044**
	One year	Mean (±SD), *n*	17.2	(±3.7),	6		
Closing pressure (mmHg)	Baseline	Mean (±SD), *n*	13.8	(±1.3),	5	−6.51 to -0.40	**0.035**
	One year	Mean (±SD), *n*	10.4	(±1.5),	5		
Visual parameters							
Friesen grade	Baseline	Mean (±SD), *n*	1.7	(±0.8),	6	−1.87 to 0.20	0.093
	One year	Mean (±SD), *n*	0.8	(±0.8),	6		
Mean RNFL worst eye (µm)	Baseline	Mean (±SD), *n*	120.0	(±43.1),	6	−60.59 to 14.25	0.172
	One year	Mean (±SD), *n*	96.8	(±9.6),	6		
MD worst eye	Baseline	Mean (±SD), *n*	−3.3	(±4.0),	6	−2.07 to 5.47	0.300
	One year	Mean (±SD), *n*	−1.6	(±1.2),	6		
Headache parameters							
Headache disability (HIT-6)	Baseline	Mean (±SD), *n*	64.7	(±6.1),	6	−17.73 to 5.40	0.229
	One year	Mean (±SD), *n*	58.5	(±14.5),	6		
Monthly headache frequency	Baseline	Mean (±SD), *n*	25.3	(±3.3),	6	−17.80 to 5.80	0.248
	One year	Mean (±SD), *n*	19.3	(±11.4),	6		
Headache severity	Baseline	Mean (±SD), *n*	4.9	(±1.6),	6	−3.08 to 1.24	0.324
	One year	Mean (±SD), *n*	4.0	(±3.5),	6		
Headache duration (hours)	Baseline	Mean (±SD), *n*	7.9	(±8.2),	6	−5.82 to -0.29	**0.036**
	One year	Mean (±SD), n	4.8	(±9.4),	6		
Analgesic use per months (days)	Baseline	Mean (±SD), *n*	12.0	(±13.2),	6	−16.95 to 7.62	0.374
	One year	Mean (±SD), *n*	7.3	(±12.0),	6		

*CSF* cerebrospinal fluid, *HIT-6* headache impact test-6 score, *NA* not applicable, *RNFL* retinal nerve fibre layer, *SD* standard deviation.

Bold values indicate significant *p* values (i.e. *p* < 0.05).

In patients, LP opening pressure was significantly higher (median = 22.07 mmHg, range = 7.36 mmHg) compared to controls (median = 16.18 mmHg, range = 7.72 mmHg), *p* < 0.0001. Frisén grade in patients ranged from 1 to 3 (mean = 1.7, SD = ± 0.7), whereas none of the controls had papilloedema. Mean worst eye RNFL was 116.4 µm (SD = ± 37.9 µm) in IIH and 99.2 µm (SD = ± 7.8 µm) in controls, *p* = 0.1766.

Headaches were noted both amongst the controls and IIH patients. All participants reported migraine like headache, except for two controls and one patient who were headache free both on headache diary and on the day of research. Medication overuse was noted in one control and four patients. There was a trend towards higher headache disability (*p* = 0.055) in IIH patients: IIH HIT-6 median = 65.0 (range = 34.0), controls HIT-6 median = 56.0 (range = 30.0). In addition, frequency of headaches was higher in IIH (*p* = 0.044): median IIH monthly headaches = 24.0 (range = 28.0), median controls’ monthly headaches = 12.0 (range = 28.0). No between-group differences were found in headache severity (*p* = 0.552) and duration (*p* = 0.284). Patients reported using analgesics for a mean 10.7 days/month (SD = ± 11.3 days/month), whereas controls for 7.1 days/month (SD = ± 7.4 days/month), *p* = 0.442.

### MRI-ICP at baseline

**Fig. 2:**
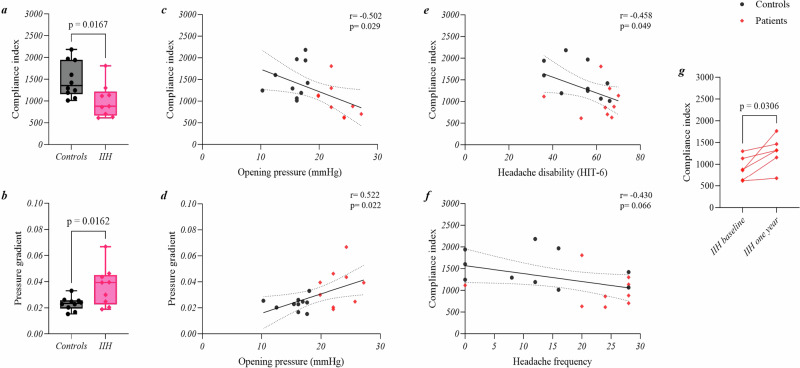
Graphs illustrating the main results of this study. **a** Intracranial compliance index is significantly reduced in IIH compared to age, sex, and BMI-matched controls at baseline. **b** Pressure gradient is significantly elevated in IIH compared to age, sex, and BMI-matched controls at baseline. **c** Significant inverse correlation between intracranial compliance index and opening pressure. **d** Significant correlation between pressure gradient and opening pressure. **e** Significant inverse correlation between headache disability (measured using the headache impact test-6 score or HIT-6) and intracranial compliance index. **f** Association between monthly frequency of headaches and intracranial compliance index. **g** Significant increase in intracranial compliance index in IIH after one year following treatment.

Resulting MRI-ICP parameters are reported in Table 2. Figure 2a illustrates that patients had significantly reduced intracranial compliance index (mean = 1006.0cc/mmHg/cm, SD = ± 384.6cc/mmHg/cm), compared to controls (mean = 1493.0cc/mmHg/cm, SD = ± 411.8cc/mmHg/cm), *p* = 0.017. In addition, MRI-measured pressure gradient was significantly higher in patients (mean=0.037 mmHg/cm, SD = ± 0.015 mmHg/cm) than in controls (mean = 0.023 mmHg/cm, SD = ± 0.005 mmHg/cm), *p* = 0.016, Fig. 2b. No significant differences were found in CSF (*p* = 0.661), arterial (*p* = 0.574), or venous (*p* = 0.842) stroke volumes; no significant between-group differences were found in heart rate (mean patients = 74.2, beats per minute, SD ± 10.8, mean controls = 72.0, SD ± 11.8, *p* = 0.68).

**Table 2:** MRI-ICP parameters at baseline and at one-year.

Cross-sectional: patients and controls						95% confidence interval	*p*
Compliance index (cc/mmHg/cm)	Controls	Mean (±SD), *n*	1493.0	(±411.8),	10	−873.90 to −99.90	**0.017**
	Patients	Mean (±SD), *n*	1006.0	(±384.6),	9		
Pressure gradient (mmHg/cm)	Controls	Mean (±SD), *n*	0.02	(±0.01),	10	0.003 to 0.02	**0.016**
	Patients	Mean (±SD), *n*	0.04	(±0.02),	9		
CSF stroke volume (cc)	Controls	Median (range), *n*	0.6	(1.0),	10	NA	0.661
	Patients	Median (range), *n*	0.6	(0.8),	9		
Arterial stroke volume (cc)	Controls	Mean (±SD), *n*	7.8	(±1.8),	10	−1.43 to 2.51	0.574
	Patients	Mean (±SD), *n*	8.3	(±2.2),	9		
Venous stroke volume (cc)	Controls	Median (range), *n*	14.3	(14.9),	10	NA	0.842
	Patients	Median (range), *n*	16.5	(10.2),	9		
Heart rate (beats per minute)	Controls	Mean (±SD), *n*	72.0	(±11.8)	10	−8.78 to 13.17	0.678
	Patients	Mean (±SD), *n*	74.2	(±10.8)	9		
Longitudinal: patients							
Compliance index (cc/mmHg/cm)	Baseline	Mean (±SD), *n*	903.8	(±273.2),	6	53.04 to 708.40	**0.031**
	One year	Mean (±SD), *n*	1285.0	(±362.8),	6		
Pressure gradient (mmHg/cm)	Baseline	Mean (±SD), *n*	0.04	(±0.02),	6	−0.02 to 0.01	0.193
	One year	Mean (±SD), *n*	0.03	(±0.01),	6		
CSF stroke volume (cc)	Baseline	Mean (±SD), *n*	0.7	(±0.3),	6	−0.18 to 0.25	0.693
	One year	Mean (±SD), *n*	0.8	(±0.4),	6		
Arterial stroke volume (cc)	Baseline	Mean (±SD), *n*	8.0	(±2.4),	6	−1.65 to 3.47	0.401
	One year	Mean (±SD), *n*	8.9	(±1.5),	6		
Venous stroke volume (cc)	Baseline	Mean (±SD), *n*	15.2	(±3.7),	6	−1.36 to 5.75	0.173
	One year	Mean (±SD), *n*	17.3	(±3.8),	6		
Heart rate (beats per minute)	Baseline	Mean (±SD), *n*	74.4	(±12.9)	6	−17.11 to 6.11	0.278
	One year	Mean (±SD), *n*	68.9	(±15.3)	6		

*CSF* cerebrospinal fluid, *NA* not applicable, *SD* standard deviation.

Bold values indicate significant *p* values (i.e. *p* < 0.05).

### MRI-ICP and clinical measures

Lumbar pressure opening pressure correlated inversely with compliance index (r = −0.502, *p* = 0.029) and directly with MRI-measured pressure gradient (r = 0.522, *p* = 0.022), Fig. 2c, d, Table 3. There were no significant associations between compliance index and optical coherence tomography RNFL (r = −0.411, *p* = 0.080), a measure of papilloedema.

**Table 3:** Regressions between clinical and MRI-ICP parameters.

Simple linear regressions			*r*	95% confidence interval	*p*
Opening pressure (mmHg)	Compliance index (cc/mmHg/cm)		−0.5019	−0.78 to −0.06	**0.029**
RNFL (µm)	Compliance index (cc/mmHg/cm)		−0.4113	−0.73 to 0.05	0.080
Headache disability (HIT-6)	Compliance index (cc/mmHg/cm)		−0.4581	−0.76 to −0.01	**0.049**
Monthly headache frequency (days)	Compliance index (cc/mmHg/cm)		−0.4304	−0.74 to 0.03	0.066
Headache severity	Compliance index (cc/mmHg/cm)		−0.1919	−0.59 to 0.29	0.431
Headache duration (hours)	Compliance index (cc/mmHg/cm)		−0.1845	−0.59 to 0.29	0.450
Opening pressure (mmHg)	MRI-pressure gradient (mmHg/cm)		0.5223	0.09 to 0.79	**0.022**

Multiple linear regressions		*VIF*	R^2^	Estimate (95% confidence interval)	*p*
Headache disability (HIT-6)	Intercept			66.56 (53.11 to 80.02)	**<0.0001**
	Compliance index (cc/mmHg/cm)	1.0	0.503	−0.013 (−0.023 to −0.003)	**0.014**
	Days analgesic used per month	1.0		0.68 (0.19 to 1.17)	**0.009**
Monthly headache frequency (days)	Intercept			27.22 (14.85 to 39.60)	**0.0003**
	Compliance index (cc/mmHg/cm)	1.0	0.448	−0.011 (−0.020 to −0.002)	**0.018**
	Days analgesic used per month	1.0		0.53 (0.08 to 0.98)	**0.025**

*HIT-6* headache impact test-6 score, *RNFL* retinal nerve fibre layer, *VIF* variance inflation factor.

Bold values indicate significant *p* values (i.e. *p* < 0.05).

Table 3 and Fig. 2e show the significant inverse correlation between compliance index and headache disability on HIT-6 (r = −0.458, *p* = 0.049). There was also a trend for an inverse association between lower compliance index and increased frequency of headaches (r = −0.430, *p* = 0.066), Fig. 2f. No correlations between compliance index and headache severity (*p* = 0.431) or duration (*p* = 0.450) were detected. When accounting for use of analgesics (Table 3), the inverse correlation between compliance index and headache disability became stronger (*p* = 0.014) and the association between headache frequency and compliance index became significant (*p* = 0.018).

### Longitudinal results

Six patients attended at the one year follow up appointment following a weight loss intervention (three had bariatric surgery and three undertook a community weight loss intervention). A significant reduction in LP opening pressure was detected (mean of differences = −5.88 mmHg, 95% confidence interval = −11.51 to −0.25 mmHg, *p* = 0.044) as well as a reduction in average headache duration (mean of differences = −3.1 h, 95% confidence interval = −5.82 to −0.29 h, *p* = 0.036), whereas there were no significant differences in other clinical and visual parameters, Table 1.

Intracranial compliance index was significantly increased at one year (mean of differences = 380.7 cc/mmHg/cm, 95% confidence interval = 53.04–708.40 cc/mmHg/cm, *p* = 0.031), with other MRI-ICP parameters remaining stable, Table 2, Fig. 2g. Intracranial compliance index improved in all patients, however, the largest change was detected in those who had undergone bariatric surgery (increase between 50% and 105%) compared to those assigned to community weight loss programmes (increase ranged from 10% to 16%).

## Discussion

To the best of our knowledge, this is the first study to apply longitudinally the non-invasive and quantitative MRI-ICP technique to measure intracranial compliance index in a carefully phenotyped cohort of women with IIH. It shows that intracranial compliance index (measured by MRI-ICP) is reduced in IIH. Furthermore, we demonstrate that intracranial compliance index is inversely proportional to LP opening pressure, correlates with headache disability, and improves at 12 months with IIH weight loss treatment which lowered ICP.

Compliance is defined as change in intracranial volume per pressure unit change and has classically been estimated from invasive intracranially-inserted ICP monitors often requiring invasive infusions [37]. Typically, the parameters used are pulse amplitude (i.e. the difference between the maximum and minimum of the pulse pressure) or features of the pressure waveforms derivable from specialised monitors [38]. The MRI-ICP technique may provide a non-invasive alternative in IIH. It is a technique based on the well-established mono-exponential relationship between changes in ICP and changes in intracranial volume [39, 40], rendering ICP inversely proportional to compliance [19]. In this study, we have shown that MRI-ICP-measured compliance index was indeed inversely associated with LP opening pressure, indicating that the mathematical relationship held true in a real-world scenario and offering reassurance on the reliability of the technique.

We have shown that there was a significant reduction in compliance index in IIH compared to age and BMI matched controls. To date, there are very few other MRI studies utilising cine phase sequences in IIH: Alperin et al. utilised phase-contrast sequences to measure arterial and venous flow rates in IIH, showing reduced venous drainage in 11 IIH patients compared to 11 controls, although compliance and stroke volumes were not calculated [27]. Another study was primarily aimed at developing a model of the cranio-spinal compliance distribution (as opposed to intracranial compliance) which was then tested in six healthy individuals and seven patients, showing reduced spinal canal compliance in IIH. No correlations with clinical measures or longitudinal changes were assessed [28]. A recent MRI study employed MR elastography and detected increased brain stiffness in IIH compared to controls [41]. Our findings are in line with previous literature utilising invasive measurements, although intracranial compliance index cannot be directly converted into elastance measured invasively, hence comparisons with current literature are only qualitative. For instance, two studies calculated elastance (a measure inversely associated with compliance) from LP derived parameters and showed it to be increased in IIH patients [42, 43]. Okon et al. estimated reduced compliance in IIH from the pressure waveforms obtained by draining CSF during an LP [44]. A study in children with IIH utilising CSF infusion tests, and two studies utilising telemetric monitoring in adult patients all reported increased pulse pressure amplitude, another surrogate measure of reduced compliance [29, 45, 46].

This imaging study also indicated that the MRI-ICP measured compliance index might have clinical relevance: compliance index correlated with headache disability and frequency of headaches, possibly reflecting a lower threshold to developing migranous headaches in IIH patients [9]. In addition, the parameter improved following weight loss intervention. Even though this study was not designed to show efficacy of the weight loss intervention on main visual and clinical parameters, the results of the main IIH:WT trial have now been published demonstrating that bariatric surgery was superior to community weight loss programmes in the treatment of women with active IIH and a BMI greater than or equal to 35 kg/m^2^ [25]. In line with those findings, a reduction in LP opening pressure paralleled by improvement in compliance index was shown in our study. Compliance index increased in all patients, with the largest increase noted in those who had received surgery. This suggests that MRI-ICP has the potential to be a sensitive non-invasive tool to monitoring disease progression or improvement with treatment.

There was variability observed, which is likely to be multifactorial. Part of the variability may be due to technical factors related to MRI hardware and acquisitions. Although there are studies that have assessed repeatability and reproducibility of phase-contrast MRI sequences [47, 48], reproducibility of the MRI-ICP technique in IIH population will need to be investigated in future dedicated studies. However, it is likely that some of the variance may be due to biological factors. Minute variations in neck position (flexion or extension) as well as hip flexion are also known to alter LP opening pressure values [49].

The main limitation of this study is the relatively small sample size which likely resulted in type two error and lack of significance in RNFL differences between patients and controls. Nonetheless, it was possible to illustrate changes in compliance index, with over 50% of patients having values below the minimum of controls. This indicates potential for the technique as a diagnostic aid, pending validation in larger cohorts. Another limitation was the use of LP opening pressure instead of intracranially-inserted ICP probe monitoring for assessment of correlations with MRI-ICP. Although ICP probe monitors would have allowed multiple measurements under different conditions, ethical concerns may be raised when considering implantation to non-IIH controls. Hence, between group comparisons would not have been feasible in this study. In addition, opening pressure is sufficient for diagnostic purposes in most clinical cases [6, 14].

In conclusion, we have shown that MRI-ICP is feasible in IIH and shows a biologically plausible compliance index reduction in IIH which might contribute to headaches disability. The technique also replicates well established physiological relations with LP opening pressure. Lastly, compliance index improves with IIH treatment, suggesting that the MRI-ICP technique might be a suitable non-invasive biomarker for IIH disease progression and severity thus guiding future research studies.

## Summary

### What was known before

1. Idiopathic intracranial hypertension (IIH) is a disease affecting women of childbearing age with obesity. It can threaten vision and causes disabling headaches.
2. Raised intracranial pressure (ICP) is one of the cardinal features of IIH and it is usually measured invasively.
3. The MRI-ICP imaging technique allows non-invasive measurement of intracranial compliance index, which could be used as a surrogate marker of raised ICP in IIH.

### What this study adds

1. The MRI-ICP technique is feasible in IIH and shows a biologically plausible reduction in compliance index in IIH compared to controls.
2. The technique also replicates well established physiological relations with LP opening pressure and indicates that compliance index inversely correlates with headaches disability.
3. Compliance index improves with IIH treatment, suggesting that the MRI-ICP technique might be a suitable non-invasive biomarker of IIH disease progression and severity.

## Data Availability

Anonymized individual participant data may be made available. Proposals should be made to the corresponding author and will be reviewed by the Birmingham Clinical Trials Unit Data Sharing Committee in discussion with the Chief Investigator. A formal Data Sharing Agreement may be required between respective organizations once release of the data are approved and before data can be released.
